# Bringing a New Flexible Mercaptoacetic Acid Linker to the Design of Coordination Polymers

**DOI:** 10.3390/polym12061329

**Published:** 2020-06-10

**Authors:** Agnieszka Ostasz, Alexander M. Kirillov

**Affiliations:** 1 Department of General and Coordination Chemistry and Crystallography, Institute of Chemical Science, Faculty of Chemistry, Maria Curie-Skłodowska University, M.C. Skłodowska Sq. 2, 20-031 Lublin, Poland; 2 Centro de Química Estrutural, Instituto Superior Técnico, Universidade de Lisboa, Av. Rovisco Pais, 1049-001 Lisbon, Portugal; 3 Research Institute of Chemistry, Peoples’ Friendship University of Russia (RUDN University), 6 Miklukho-Maklaya st., 117198 Moscow, Russia

**Keywords:** coordination polymers (CPs), metal-organic frameworks (MOFs), hydrothermal synthesis, precipitation synthesis, thermal analysis, topological analysis, solid-state phase transitions

## Abstract

Two new 3D coordination polymers (CPs), formulated as [Zn(*p*-XBT)]_n_ (**1H**) and [Cd(*p*-XBT)]_n_ (**2H**), were assembled from a virtually unexplored *p*-xylylene-bis(2-mercaptoacetic) acid linker (*p*-XBTA) and characterized by infrared spectroscopy (FTIR), powder X-ray diffraction (PXRD), thermal analysis methods (TG-DSC, TG-FTIR), single-crystal X-ray diffraction, and topological analysis. Two different synthetic strategies were explored, namely the precipitation (**P**) and hydrothermal (**H**) methods, resulting in a Zn(II) derivative [Zn(*p*-XBT)·H_2_O]_n_ (**1P**) and its dehydrated analogue [Zn(*p*-XBT)]_n_ (**1H**), respectively. In the Cd(II)-containing system, the same [Cd(*p*-XBT)]_n_ (**2P** = **2H**) products were generated by both synthetic methods. Upon dehydration, **1P** undergoes a “crystal-to-crystal” phase transition in the 170−185 °C temperature range, producing an anhydrous polycrystalline sample (**1H**). Both CPs **1H** and **2H** are isostructural and feature polymeric 3D metal-organic nets of the **cds** topological type, which are driven by the 4-linked metal and *p*-XBT^2−^ nodes. These compounds represent unique examples of coordination polymers derived from *p*-xylylene-bis(2-mercaptoacetic) acid, thus opening up the use of this flexible S,O-heterodonor building block in the design of polymeric metal-organic architectures.

## 1. Introduction

Coordination polymers (CPs) have attracted an extraordinary interest in many branches of modern chemistry owing to their porosity, uniform pore sizes, very high surface areas, and finely tunable functional properties [1,2,3]. In contrast to the highly prolific production of diverse CPs based on rigid aromatic carboxylate ligands, the design and synthesis of coordination polymers, incorporating flexible and heterodonor-containing building blocks, have so far attracted less attention [4,5]. Among such types of linker ligands, compounds containing both thio- and carboxylate functional groups have remained poorly explored in the design of polymeric metal-organic architectures. Following our interest in applying novel and flexible multifunctional building blocks to the preparation of metal-organic networks [6,7,8], herein we have focused our attention on *p*-xylylene-bis(2-mercaptoacetic) acid (*p*-XBTA). This organic building block bears a xylylene core and contains two carboxylic acid groups as well as flexible thioaliphatic spacers −CH_2_–S-CH_2_−. The mercaptoacetic moieties are strongly twisted away from the plane of the xylylene unit due to the *sp*^3^ hybridization of sulfur and methylene carbon atoms. As such, this is a flexible and very interesting ligand for the construction of coordination polymers which has never been explored, as confirmed by a search of the CSD (Cambridge Structural Database) [9].

**Scheme 1:**
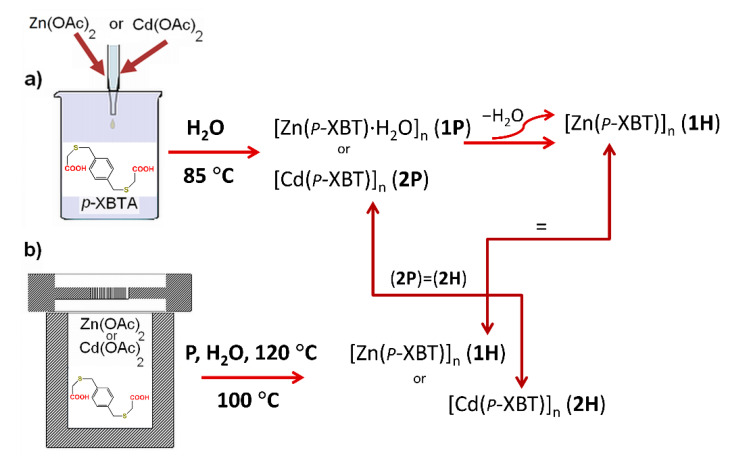
Schematic representation of (**a**) reverse precipitation (for **1P** and **2P**) and (**b**) hydrothermal (for **1H** and **2H**) synthesis. *p*-XBTA: *p*-xylylene-bis(2-mercaptoacetic) acid.

Hence, the main aim of the present work was to synthesize new coordination polymers derived from *p*-XBTA by exploring different hydrothermal and reverse precipitation methods in aqueous medium. These methods could allow to cope with sparingly soluble ligands in water and to obtain pure crystalline products [10,11,12]. This study thus reports on the preparation, complete characterization, crystal structures, spectroscopic features, topological classification, and thermal behavior of two new polymeric 3D metal-organic architectures driven by flexible *p*-XBT^2−^ linkers. Two series of products generated hydrothermally (series **H**) and by precipitation method (series **P**) were isolated, namely, [Zn(*p*-XBT)]_n_ (**1H**), [Zn(*p*-XBT)·H_2_O]_n_ (**1P**), and [Cd(p-XBT)]_n_ (**2H** = **2P**) (Scheme 1). As confirmed by a search of the CSD, these derivatives constitute unique examples of coordination compounds assembled from *p*-xylylene-bis(2-mercaptoacetic) acid, thus introducing this flexible S,O-heterodonor linker into research on coordination polymers.

## 2. Experimental

### 2.1. Materials and Methods

*p*-Xylylene-bis(2-mercaptoacetic) acid was synthesized as described in our previous work [6]. Zn(II) and Cd(II) acetate hydrates were purchased from POCH (Gliwice, Poland) and used without further purification. ATR-FTIR spectra were recorded over the range of 4000–600 cm^−1^ on a Nicolet 6700 FTIR spectrometer (Thermo Electron Corporation, Madison, WI, USA) equipped with a universal ATR attachment with a ZnSe crystal. The PXRD and VT PXRD patterns were recorded on a PANanalytical Empyrean automated diffractometer (Panalytical, Almelo, Netherlands) with Bragg–Brentano geometry using Cu Kα radiation (λ = 1.5406 Å). The intensity data were captured with a PIXcel detector with 2θ scans performed in the range of 4–60°. The thermal behavior of the compounds was investigated on a Setsys 16/18 thermal analyzer (Setaram, Caluire, France), registering the TG, DSC, and DTG curves. The samples (5 mg) were heated in a ceramic crucible in the temperature range of 30–1200 °C in a flowing air atmosphere (V = 1 dm^3^ h^−1^) with a heating rate of 10 °C min^−1^. The TG-FTIR measurements were performed on a Q5000 TA apparatus (TA Instruments, New Castle, DE, USA) coupled with the Nicolet 6700 spectrophotometer (Thermo Scientific, USA). Samples of about 6−10 mg were heated up to 1000 °C at a heating rate of 20 °C min^−1^ in a flowing nitrogen atmosphere (25 mL∙min^−1^).

### 2.2. Precipitation Synthesis of **1P** and **2P**

Precipitation synthesis was performed in the EasyMaxTM reactor (Mettler Toledo, Switzerland) with the internal measurements of the reaction temperature (100 mL reactor volume). The total synthesis time was 3.5 h. For these experiments, an aqueous solution of Zn(II) or Cd(II) acetate (30 mL, 0.9 mmol, pH = 6.1) was added to the reactor through the dosing system. Dosing of an aqueous solution of metal(II) acetate was made with a flow rate of 0.2 mL min^−1^ after stabilizing the reaction temperature at 85 °C to get a complete dissolution of *p*-xylylene-bis(2-mercaptoacetic) acid (30 mL, 0.9 mmol, pH = 4) in water (the acid is insoluble at room temperature). The stirring speed (300 rpm) and flow rate of dosing solution were controlled by the external software. After completing the synthesis, the reaction solutions were filtered off and the solids were dried in air, resulting in crystalline powders of the coordination polymers [Zn(*p*-XBT)·H_2_O]_n_ (**1P**) and [Cd(*p*-XBT)]_n_ (**2P**), isolated in 86% and 79% yields, respectively.

### 2.3. Hydrothermal Synthesis of **1H** and **2H**

An aqueous solution of Zn(II) or Cd(II) acetate (15 mL, 0.9 mmol) was mixed with an aqueous suspension of *p*-xylylene-bis(2-mercaptoacetic) acid (30 mL, 0.9 mmol, pH = 4). Complete dissolution of the acid was achieved by stirring the obtained suspension for 20 min at 85 °C. Then, the reaction mixtures were heated in 100 mL stainless steel reactors with a Teflon-lined autoclave under autogenous pressure at 100 °C for five days. After completion of the reaction, the autoclaves were slowly cooled down to room temperature, producing crystalline solids. These were isolated by filtration and dried in air to give samples of [Zn(*p*-XBT)]_n_ (**1H**) and [Cd(*p*-XBT)]_n_ (**2H**), including single crystals suitable for X-ray diffraction. Yields were 72% (**1H**) and 68% (**2H**).

### 2.4. Single-Crystal X-ray Diffraction

The X-ray diffraction measurements were performed on an Oxford Diffraction Xcalibur CCD diffractometer (Oxford Diffraction Ltd., Abingdon, UK) with graphite-monochromated Mo K_α_ radiation (λ = 0.71073 Å). The data sets were collected at 100 K using the ω scan technique, with an angular scan width of 1.0°. The CrysAlis CCD and CrysAlis Red [13] programs were used for data collection, cell refinement, and data reduction. A multi-scan absorption correction was applied. The structures were solved via direct methods using SHELXS-97 [14] and refined via the full-matrix least squares on *F*^2^ using SHELXL-97 [14]. Calculations were performed with the WinGX package [15]. All non-H atoms were refined with anisotropic displacement parameters. All H atoms were located within the difference Fourier maps and refined isotropically. The summary of crystal data, experimental details, and refinement results is given in Table 1. The selected bond distances and angles are presented in Table S1 (Supplementary Materials). The molecular plots were drawn using Mercury [16], Diamond [17], or Topos [18,19] software. Topological analysis of the structures was carried out by applying the concept of the underlying network [18,19]. Such networks were constructed by reducing the *p*-XBT^2−^ linkers to their centroids. The CIF files can be retrieved from the Cambridge Crystallographic Data Centre (CCDC 1918912-1918913).

## 3. Results and Discussion

### 3.1. FTIR Analysis

**Figure 1:**
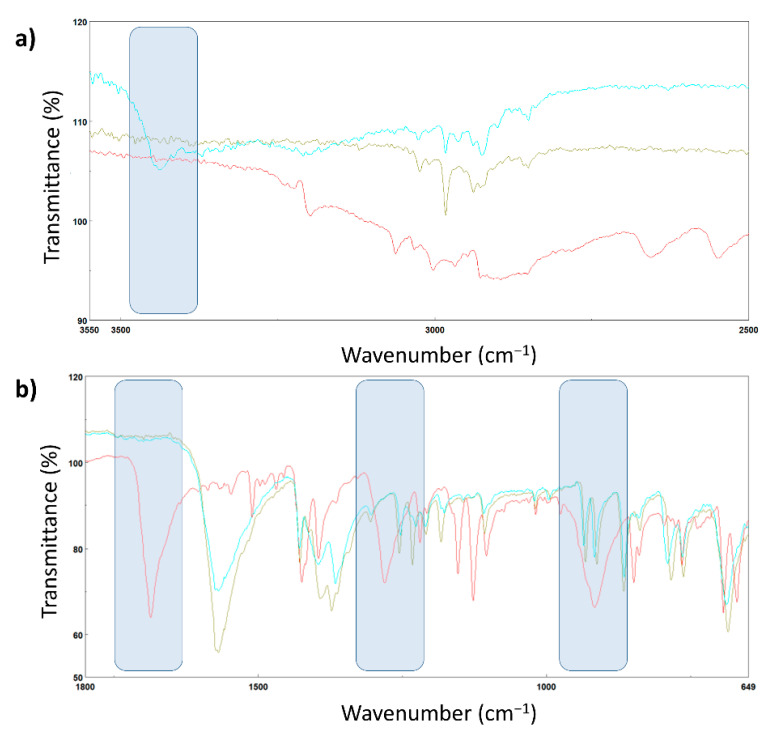
(**a**) ATR-FTIR spectra of **1P** (cyan), **2H** (green), and *p*-XBTA (red) in the 3500−2500 cm^−1^ region. (**b**) ATR-FTIR spectra of **1H** (cyan), **2H** (green), and *p*-XBTA (red) in the 1800−650 cm^−1^ region.

The FTIR spectra of the Zn(II) and Cd(II) coordination polymers (Figure 1a,b) obtained by different methods show almost the same band position, thus indicating a similarity of their structures. Characteristic bands for the asymmetric ν_as_(COO⁻) and symmetric ν_s_(COO⁻) stretching vibrations of the carboxylate groups were observed in the 1565–1570 and 1362–1368 cm^−1^ regions, respectively [20]. A shifted position of these bands relative to *p*-XBTA confirms a coordination of the ligand. Besides this, there are no bands of the COOH groups at 1686 cm^−1^ ν(C=O), 1280 cm^−1^ ν(C–OH), or 916 cm^−1^ γ(O–H) in the spectra of CPs (Figure 1b) [21]. In addition, the spectrum of **1P** reveals a broad band at 3435 cm^−1^ due to the presence of crystallization water in the structure (Figure 1a).

### 3.2. Crystal Structures

The single-crystal X-ray diffraction analysis of **1H** and **2H** reveals that both compounds feature polymeric 3D metal-organic structures, driven by the µ_4_-*p*-XBT^2−^ linkers. Both compounds were isostructural (Table 1), and thus only the Zn(II) derivative [Zn(p-XBT)]_n_ (**1H**) is described in detail as an example.

**Figure 2:**
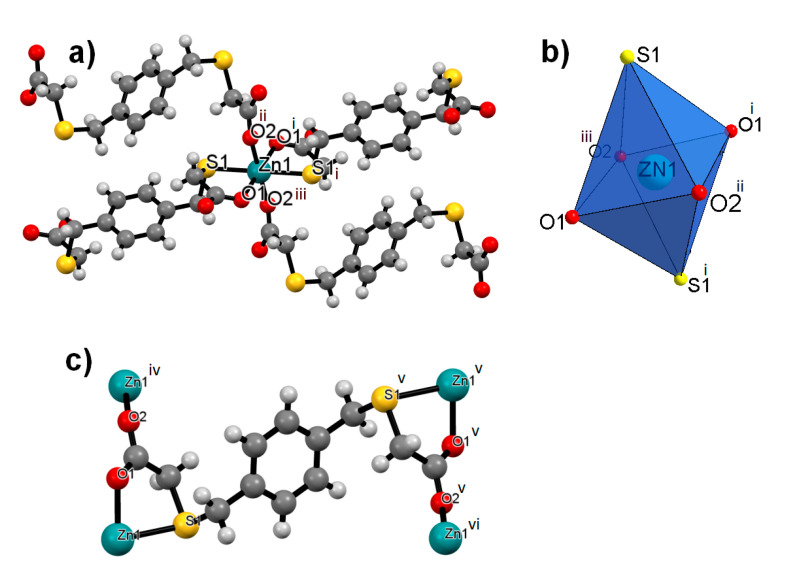
Structural fragments of **1H**. Coordination environment (**a**) and polyhedron (**b**) of Zn1 center. (**c**) Coordination mode of µ_4_-*p*-XBT^2−^. Symmetry codes: (i) -x, -y, -z; (ii) x, -1/2-y, -1/2+z; (iii) -x, 1/2+y, 1/2-z; (iv) -x, -1/2+y, 1/2-z; (v) 1-x, -y, 1-z; (vi) 1-x, 1/2+y, 1/2-z.

The compound **1H** crystallizes in the monoclinic space group P2_1_/c with one-half metal ion and one-half doubly deprotonated µ_4_-*p*-XBT^2−^ linker in the asymmetric unit (both Zn1 and µ_4_-*p*-XBT^2−^ are located on an inversion center). The 6-coordinate Zn1 atoms are bound by four oxygen (O1, O2) and two sulfur (S1) atoms from four µ_4_-*p*-XBT^2−^ ligands forming a slightly distorted octahedral environment (Figure 2). The Zn−O distances range from 2.045(1) to 2.134(1) Å, while the Zn−S distance is 2.524(1) Å. The comparison between the geometric parameters of **1H** and **2H** shows that the Cd−O (2.240(2) and 2.304(2) Å) and Cd−S (2.686(1) Å) distances in the Cd(II) product are expectedly longer than those in the Zn(II) compound. However, all bond lengths are similar to those found in related derivatives [22,23,24,25,26,27]. The *p*-XBTA^2−^ moiety acts as a µ_4_-linker and an S_2_,O_4_-hexadentate ligand (Figure 2c). The carboxylate groups adopt a bridging anti-anti coordination mode. Additionally, the carboxylate oxygen atom (O1) and sulfur atom (S1) chelate the Zn1 atoms, resulting in the formation of five-membered rings (S1-Zn1-O1-C1-C2) (Figure 2c).

**Figure 3:**
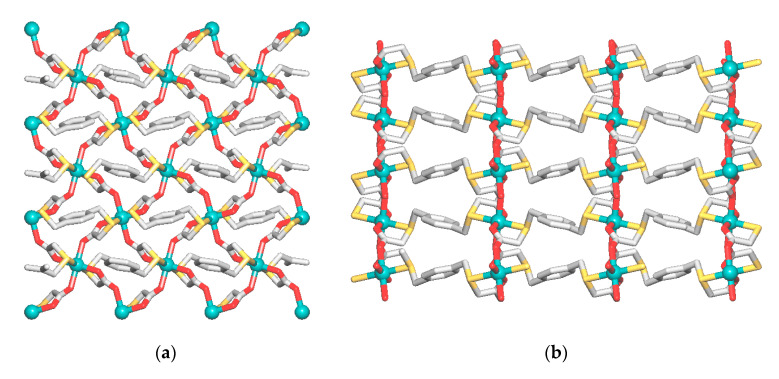

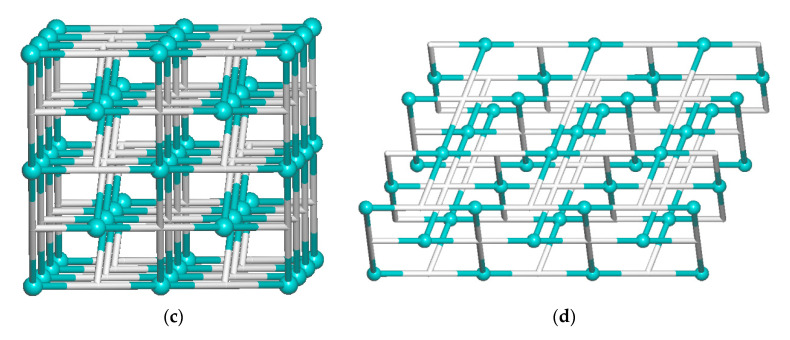
Structural and topological representations of polymeric metal-organic networks in **1H**. (**a**,**b**) 3D coordination polymer structure seen along the *a* (**a**) and *c* (**b**) axis; H atoms are omitted; Zn (turquoise balls), O (red), S (yellow), C (gray). (**c**,**d**) Perspective topological views of a simplified 3D network displaying a uninodal 4-connected net with a **cds** (CdSO_4_) topology. Rotated views along the *a* (**c**) and *c* (**d**) axis; color codes: 4-connected Zn nodes (turquoise balls), centroids of 4-connected µ_4_-*p*-XBT^2−^ nodes (gray).

An overall 3D MOF structure for **1H** was achieved via a repeating linkage of Zn1 atoms by the µ_4_-*p*-XBT^2^^−^ blocks (Figure 3a,b). Herein, a sixteen-membered cyclic motif defined by four Zn1 atoms and carboxylate groups can be identified (Figure S2). The non-covalent forces stabilize the 3D network, namely some weak C_sp3_-H∙∙∙π_ar_ and C_sp3_-H∙∙∙S interactions occurring between the adjacent 2D layer motifs of **1H**. To get a better understanding of the metal-organic network in **1H**, we simplified it (Figure 3c,d) and carried out its topological classification. From a topological viewpoint, both the Zn1 atoms and µ_4_-*p*-XBTA^2^^−^ linkers can be considered as 4-connected nodes that are topologically equivalent. Classification of the resulting net reveals a uninodal 4-connected framework with a **cds** (CdSO_4_) topology and a point symbol of (6^5^.8). Compound **2H** features the same type of topology.

### 3.3. Powder X-ray Diffraction

**Figure 4:**
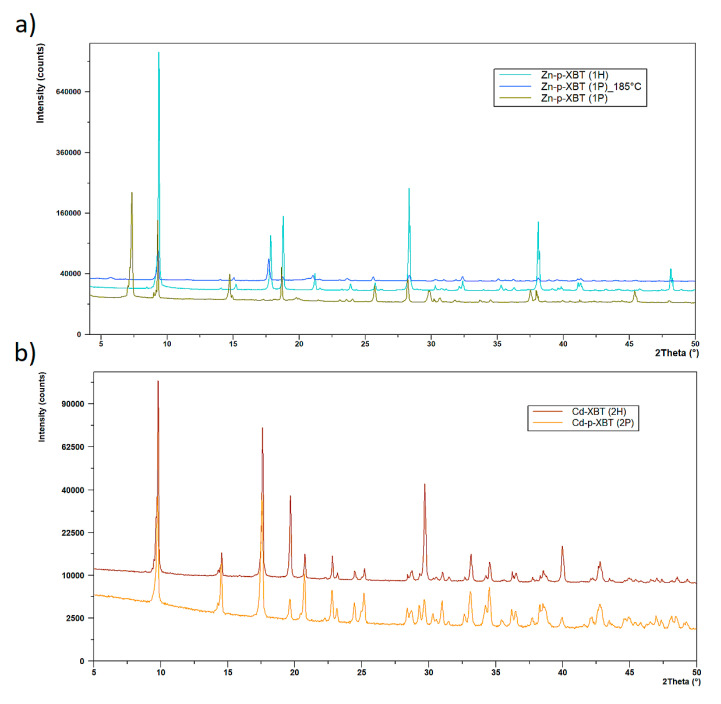
PXRD patterns: (**a**) **1H** and **1P** at 25 °C, and **1P** after heating to 185 °C; (**b**) **2H** and **2P** at 25 °C.

**Figure 5:**
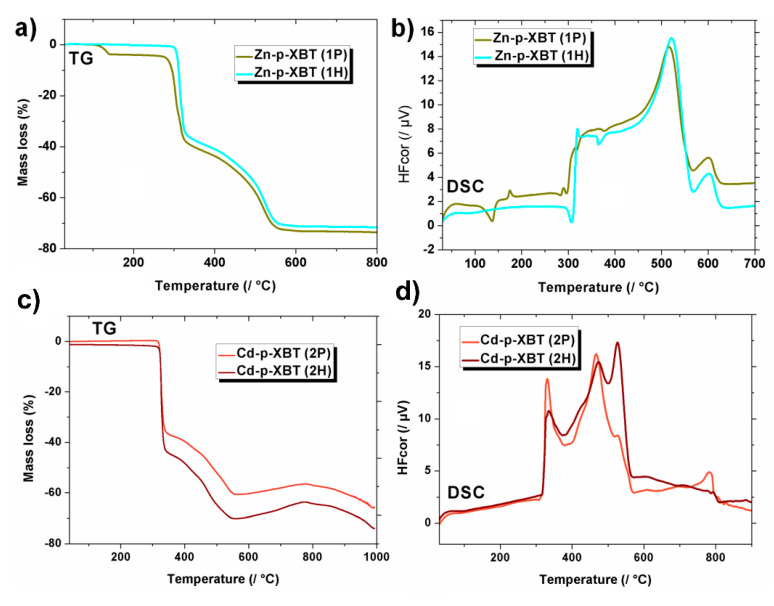
TG and DSC curves of **1H** and **2H** (**a**,**b**); **1P** and **2P** (**c**,**d**).

The PXRD analysis reveals that all compounds are crystalline (Figure 4). The PXRD patterns of **1H** and **1P** show peak positions that are mostly compatible with each other. However, the pattern of **1P** exhibits new characteristic peaks at the 2θ values of 7.4 and 19.9° that indicate some difference between **1P** and **1H** (Figure 4a), namely due to the presence of a water molecule in **1P**. In contrast, relating to the Cd(II) samples **2H** and **2P**, the PXRD data are essentially similar (Figure 4b), confirming that they have the same structure. The PXRD pattern of **1P** after heating to 185 °C was also measured (Figure 4a). The diffraction pattern of **1P** changes on heating due to a phase transition caused by dehydration. The dehydration process starts at 95 °C, as confirmed by the TG-DSC data. Upon further heating to 185 °C, some peaks disappear (e.g., at 2θ value of 7.4°), while other signals shift slightly (e.g., at the 2θ value of 14.8°) and new peaks appear (e.g., at 2θ value of 21.0°). Moreover, the intensity of most PXRD signals decreases, which indicates a partial loss of crystallinity. The VT XRPD experiment (Figure 4a) confirms the phase transition that is also visible on the DSC curve of **1P** at 170−185 °C (Figure 5b), leading to an anhydrous polycrystalline form identical to **1H**.

### 3.4. Thermal Analysis

#### 3.4.1. TG-DSC Analysis

The thermal stability and decomposition pathway of the obtained compounds was investigated using thermogravimetric analysis (TG) and differential scanning calorimetry (DSC) in air. The TG and DSC curves for **1H** reveal a multi-step decomposition accompanied by two strong exothermic effects (Figure 5a,b). The TG analysis of **1P** shows that the sample releases water molecules between ca. 110 and 158 °C (found mass loss of 4.0%; calculated at 4.8%). Then, the TG curve of a dehydrated solid shows a plateau up to 260 °C. At the same time, the DSC analysis of **1P** discloses an endothermic effect associated with the dehydration process. In the 170−185 °C temperature range, the presence of an exothermic effect (ΔH = 8.96 J/g) suggests the “crystal-to-crystal” phase transformation of **1P** to **1H** (Figure S5). This is visible on the DSC curve of **1P** at 170–185 °C (Figure 5b and Figure S5), resulting in an anhydrous polycrystalline form identical to **1H**. After this phase transition, the TG curve of **1P** reveals a long plateau before the beginning of the decomposition process at 260 °C (Figure 5a), which then produces ZnO at 700 °C (found mass loss 75.2%; calculated mass loss 77.8%) as a final decomposition product (Figure S1).

As attested by the TG-DSC analysis of the Cd(II) samples **2P** and **2H**, this compound is essentially stable up to 310 °C (Figure 5c). Then, a multistep decomposition of organic ligand begins as evidenced by a number of exothermic effects in the 310–580 °C temperature interval (Figure 5d). Further heating leads to a slight mass increase, followed by a mass loss at temperatures above 780 °C. Herein, the absence of a plateau might be associated to reactions with oxygen (air flow atmosphere) or gas adsorption. Cadmium(II) oxide is an expected decomposition product at 1000 °C (found mass loss 69.5%, avg. for **2P** and **2H**; calculated mass loss 67.6%).

#### 3.4.2. TG-FTIR Analysis

**Figure 6:**
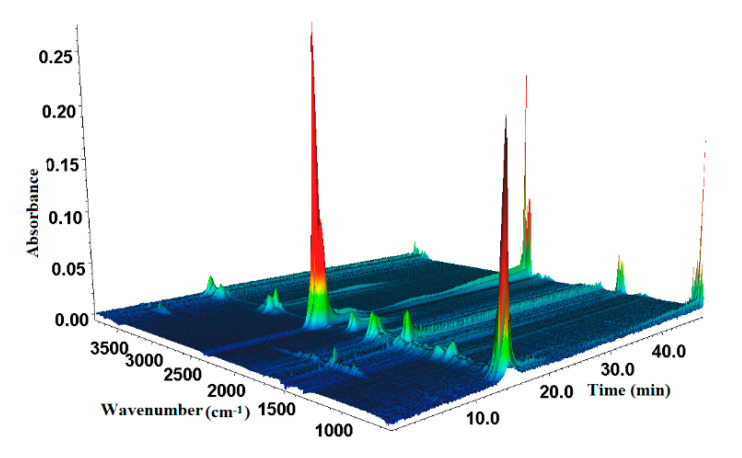
TG-FTIR stacked plot of **1P** (N_2_ atmosphere, 20 °C min^−1^ heating rate).

Simultaneous TG analysis under nitrogen atmosphere coupled with FTIR spectrophotometric analysis allows for the determination of the decomposition pathway and identification of volatile degradation products. Analysis of the volatile products released during the heating of the compound **1P** indicates that the thermal decomposition proceeds in two steps. The stacked plot of the FTIR spectra of the evolved gases for **1P** is given in Figure 6. The first step concerns the elimination of the water molecule. The FTIR spectra recorded in the temperature range of 30–200 °C show relatively weak bands in the 4000−3500 and 1800–1300 cm^−1^ regions, assigned to the stretching and deformation vibrations of water OH groups, respectively. These bands correspond to the loss of a water molecule (at about 153 °C), though only in the case of **1P**. Further heating leads to the evolution of volatile products due to a thermal decomposition of organic ligand. At about 336 °C, the FTIR spectra show bands that might correspond to carbon disulfide (CS_2_) (1550–1450 cm^−1^, ν_C=S_), carbonyl sulfide (COS) (2100–2000 cm^−1^, ν_C=O_), acetic acid (1800–1700 cm^−1^, ν_C=O_), and *p*-xylene (3150−2800, 1550−1450, 1150−1050, and 850−750 cm^−1^) (Figures S3 and S4) [28,29,30]. The FTIR spectrum of this hydrocarbon is characterized by the bands at 3121, 3060, and 2875 cm^−1^ derived from the stretching vibrations of CH_3_ and C_Ar_−H groups of *p*-substituted benzene rings. Additionally, strong bands at 1547 and 807 cm^−1^ are observed due to the stretching C_Ar_−C_Ar_ vibrations and the in-plane and out-of-plane C_Ar_−H bending vibrations. At about 400 °C, in an air-free atmosphere, there is an evolution of carbon monoxide CO (2250–2050 cm^−1^, ν_CO_) and sulfur dioxide SO_2_ (1400–1300 cm^−1^, ν_S=O_) [31,32,33] (Figure S3).

## 4. Conclusions

Herein, we used for the first time a novel and flexible mercaptoacetate building block, *p*-xylylene-bis(2-mercaptoacetic) acid, to construct two new 3D coordination polymers [Zn(*p*-XBT)]_n_ (**1H**) and [Cd(*p*-XBT)]_n_ (**2H**). These were prepared using a hydrothermal method and reverse precipitation synthesis. In particular, the reverse precipitation strategy used for **1P** and **2P** represents an attractive and still poorly explored approach toward the aqueous-medium synthesis of coordination polymers without the need for prior transformation of an insoluble linker to its soluble salt. Regardless of the different synthesis conditions (i.e., hydrothermal and precipitation methods), the isostructural Zn(II) and Cd(II) products were generated and completely characterized, revealing the 3D metal-organic networks of a **cds** topological type. Besides this, the dehydration process of the Zn(II) derivative [Zn(*p*-XBT)·H_2_O]_n_ **1P** was studied in detail using different methods, indicating that the present compound undergoes a “crystal-to-crystal” phase transition in the temperature range of 170−185 °C, producing an anhydrous polycrystalline sample which is structurally similar to **1H**. Decomposition products of **1P** were also investigated using a combined TG-FTIR method.

By reporting the unique examples of coordination polymers derived from *p*-xylylene-bis(2-mercaptoacetic) acid, the present work also contributes to widening the family of multifunctional building blocks [34,35,36,37,38] in the assembly of new polymeric metal-organic architectures. We believe that the present study will motivate further research in exploring this and related types of flexible building blocks for the synthesis of coordination polymers with a diversity of topologies, functional properties, and applications [39,40].

## Figures and Tables

**Table 1:** Summary of crystallographic data and refinement details for **1H** and **2H**.

Compound	[Zn(*p*-XBT)]_n_ (1H)	[Cd(*p*-XBT)]_n_ (2H)
Chemical formula	C_12_H_12_O_4_S_2_Zn	C_12_H_12_O_4_S_2_Cd
Formula weight	349.71	396.74
Crystal system	monoclinic	monoclinic
Space group	*P*2_1_/c	*P*2_1_/c
*a* (Å)	9.4845(6)	8.9996(5)
*b* (Å)	8.3523(8)	8.5045(11)
*c* (Å)	7.9804(4)	8.6568(6)
*β* (°)	90.502(5)	90.454(6)
*V* (Å^3^)	632.16(8)	662.55(10)
Z	2	2
*T* (K)	100(2)	100(2)
Color and shape	colorless plate	colorless plate
*D*_calc_ (g cm^−3^)	1.837	1.989
*μ* (mm^−1^)	2.278	1.968
Refl. collected	2546	2795
Refl. unique	1444	1507
*R*_(int)_	0.0281	0.0285
Refl. obs. (*I* > 2σ(*I*))	1259	1253
Refined parameters	112	112
GooF on *F*^2^	0.995	0.973
*R*_1_ (*I* > 2σ(*I*))	0.0288	0.0272
*wR*_2_ (all data)	0.0705	0.0625

## Supplementary Materials

The following are available online at https://www.mdpi.com/2073-4360/12/6/1329/s1, Figure S1: PXRD pattern of **1H** after decomposition, Figure S2: Additional crystal packing patterns of **1H**, Figures S3 and S4: FTIR spectra of gaseous products formed during the decomposition of **1P**, Figure S5: DSC plots of **1P** and **1H**, Table S1: Selected structural parameters for **1H** and **2H**.
