# Supplementary material for: Bringing a New Flexible Mercaptoacetic Acid Linker to the Design of Coordination Polymers
Source: Polymers (Basel). 2020 Jun 10;12(6):1329. doi: 10.3390/polym12061329 (PMC7361794; doi:10.3390/polym12061329)
Supplement: Supplementary file 1 [file polymers-12-01329-s001.pdf]

Supplementary Materials

Article

Bringing a New Flexible Mercaptoacetic Acid Linker to the Design of Coordination Polymers

Agnieszka Ostasz [1,*] and Alexander M. Kirillov [2,3,*]

[1] Department of General and Coordination Chemistry and Crystallography, Institute of Chemical Science, Faculty of Chemistry, Maria Curie-Skłodowska University, M.C. Skłodowska Sq. 2, 20-031 Lublin, Poland.
[2] Centro de Química Estrutural, Instituto Superior Técnico, Universidade de Lisboa, Av. Rovisco Pais, 1049-001, Lisbon, Portugal.
[3] Research Institute of Chemistry, Peoples' Friendship University of Russia (RUDN University), 6 Miklukho-Maklaya st., Moscow, 117198, Russian Federation.
* Correspondence: a.ostasz@poczta.umcs.lublin.pl (A.O.), kirillov@tecnico.ulisboa.pt (A.M.K.); Tel.: +48 81 537 57 58 (A.O.)

Received: 20 May 2020; Accepted: 4 June 2020; Published: date

Supplementary Materials: The following are available online at www.mdpi.com/xxx/s1, Figure S1: PXRD pattern of **1H** after decomposition, Figure S2: Additional crystal packing patterns of **1H**, Figures S3 and S4: FTIR spectra of gaseous products formed during the decomposition of **1P**, Figure S5: DSC plots of **1P** and **1H**, Table S1: Selected structural parameters for **1H** and **2H**.

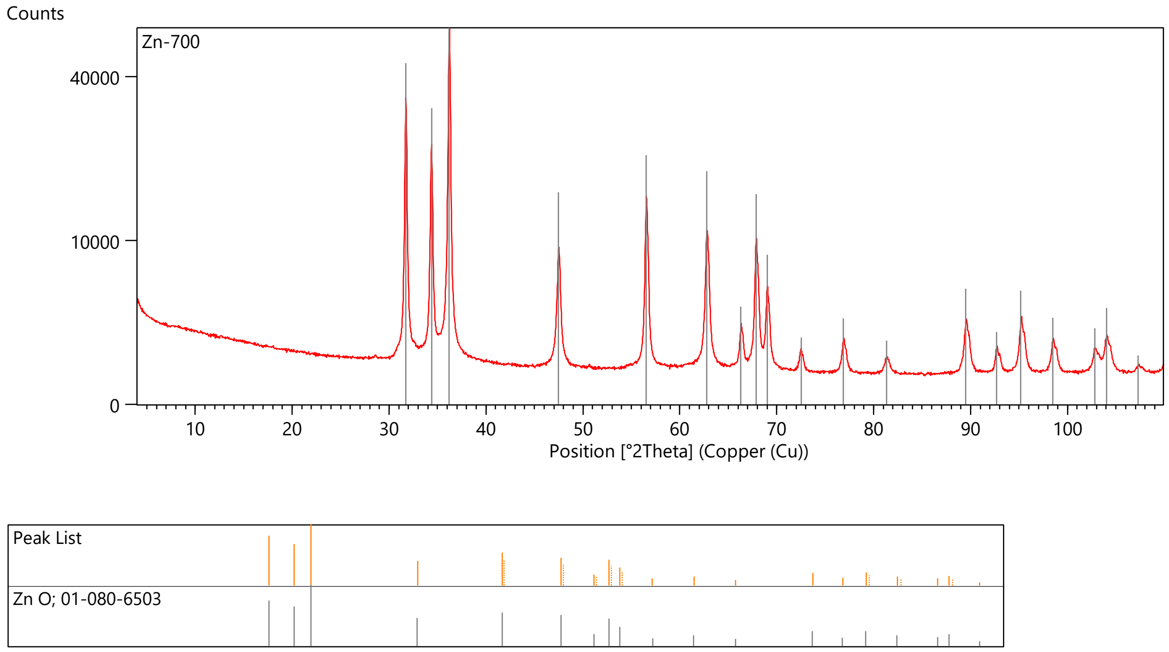

Visible	Ref. Code	Compound Name	Chemical Formula
	01-080-6503	Zinc Oxide	Zn O

Phase Zinc Oxide

Weight fraction/ %100.0

Figure S1. PXRD pattern of final residue of **Zn-*p*-XBT (1H)** after the decomposition process obtained at 700 °C (the resulting decomposition product is ZnO).

Table S1. Selected bond lengths (Å) and bond angles (°) for coordination polymers of **1H** and **2H**.

Zn1 O1^{ii}	2.0445(14)	Cd1 O1	2.240(2)
Zn1 O2^{iii}	2.1340(14)	Cd1 O2^{i}	2.304(2)
Zn1 S1^{ii}	2.5236(5)	Cd1 S1	2.6859(7)
C1 O2	1.257(2)	C1 O1	1.258(3)
C1 O1	1.265(2)	C1 O2	1.258(3)
C1 C2	1.532(3)	C1 C2	1.531(4)
C2 S1	1.800(2)	C2 S1	1.807(3)
C2 H21	0.91(3)	C2 H21	0.95(3)
C2 H22	0.92(2)	C2 H22	0.96(3)
C3 C4	1.505(3)	C3 C4	1.502(4)
O1 Zn1 O1^{ii}	180.0	O1 Cd1 O1^{iv}	180.0
O1 Zn1 O2^{iii}	86.68(6)	O1 Cd1 O2^{v}	91.79(8)
O1 Zn1 O2^{i}	93.32(6)	O1^{iv} Cd1 O2^{v}	88.21(8)
O1^{ii} Zn1 O2^{i}	86.68(6)	O1 Cd1 O2^{vi}	88.21(8)
O2^{iii} Zn1 O2^{i}	180.00(8)	O2^{v} Cd1 O2^{vi}	180.00(15)
O1 Zn1 S1^{ii}	98.58(4)	O1 Cd1 S1	76.67(5)
O1^{ii} Zn1 S1^{ii}	81.42(4)	O1^{iv} Cd1 S1	103.33(5)
O2^{iii} Zn1 S1^{ii}	91.25(4)	O2^{v} Cd1 S1	94.37(5)
O2^{i} Zn1 S1^{iii}	88.75(4)	O2^{vi} Cd1 S1	85.63(5)
O1 Zn1 S1	81.42(4)	O1 Cd1 S1^{iv}	103.33(5)
O1^{ii} Zn1 S1	98.58(4)	O1^{iv} Cd1 S1^{iv}	76.67(5)
S1^{ii} Zn1 S1	180.00(3)	S1 Cd1 S1^{iv}	180.00(1)

Symmetry transformations used to generate equivalent atoms: (i)-x, y+1/2, -z+1/2 ; (ii) -x, -y, -z; (iii) x, -y-1/2, z-1/2; (iv) -x, -y,-z+1; (v) -x, y-1/2, -z+1/2; (vi) x, -y+1/2, z+1/2

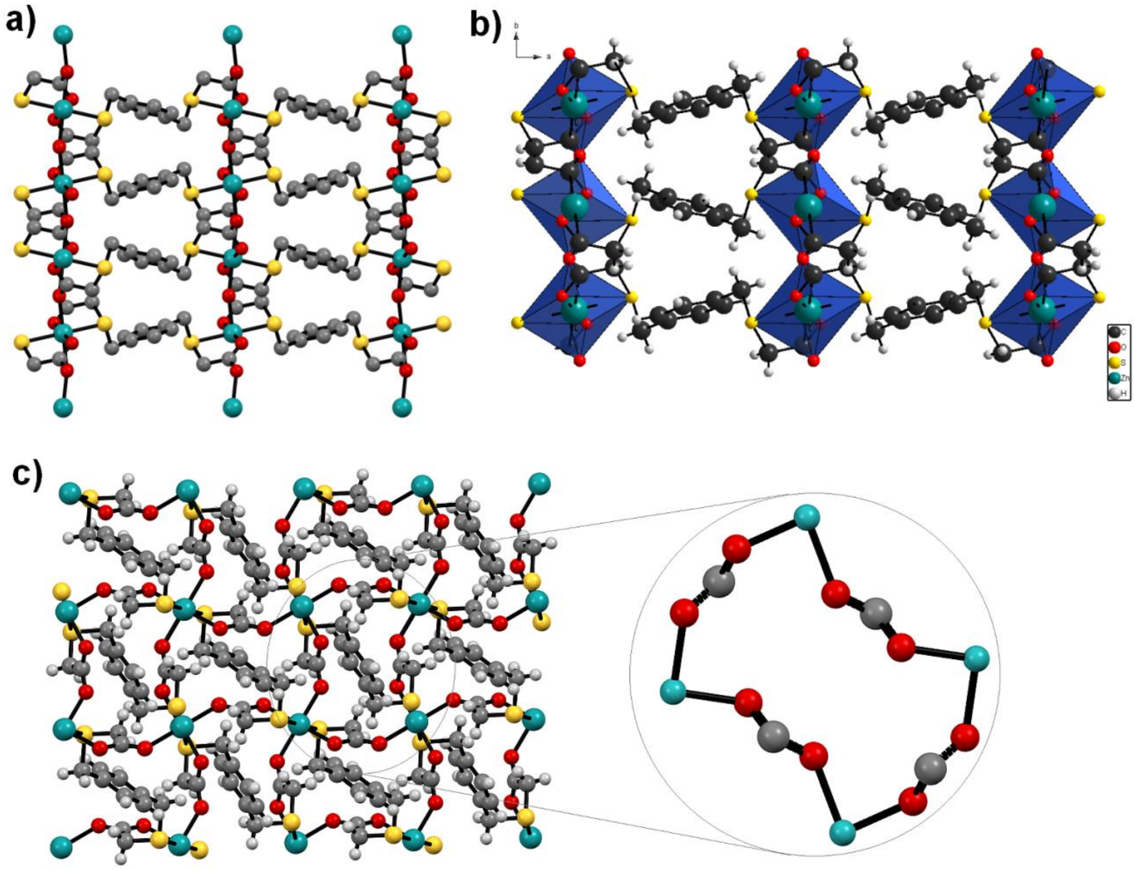

Figure S2. Crystal packing diagrams of **1H**. (a) View along the *c* axis. **(b)** Network with the polyhedral representation of Zn centers. **(b)** View along the *a* axis.

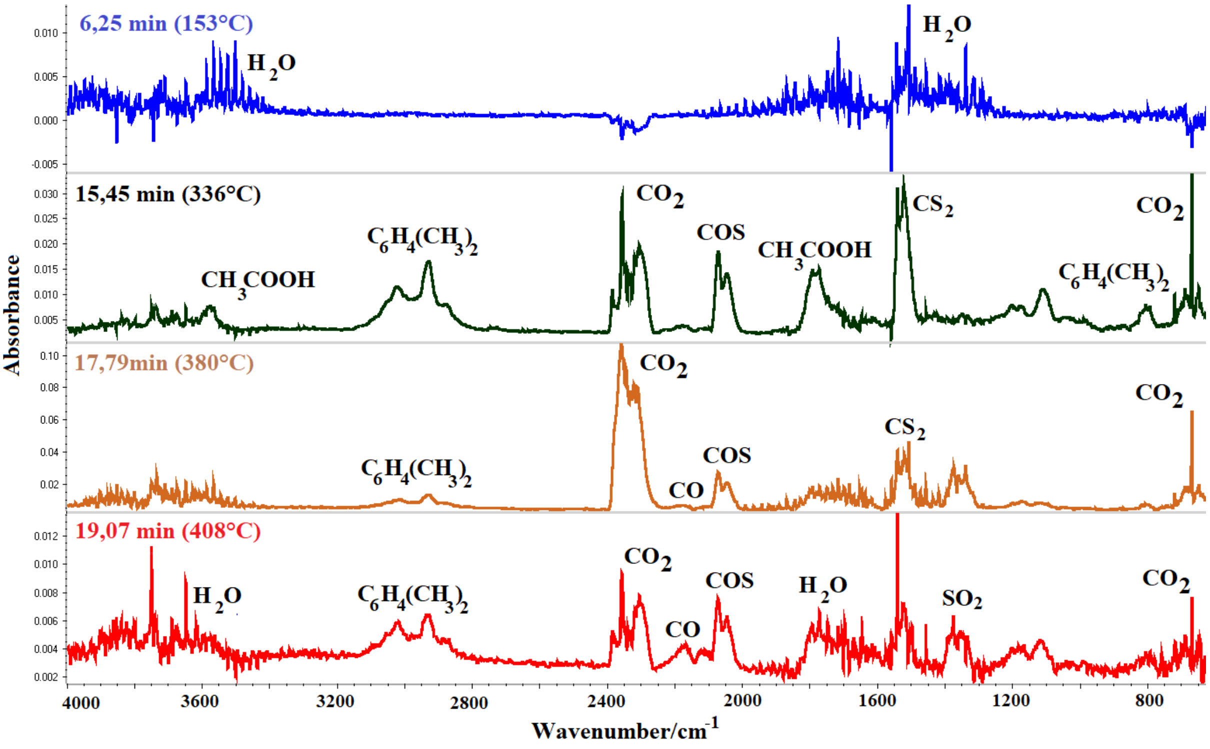

Figure S3. FTIR spectra of gaseous products obtained during the decomposition of **1P**.

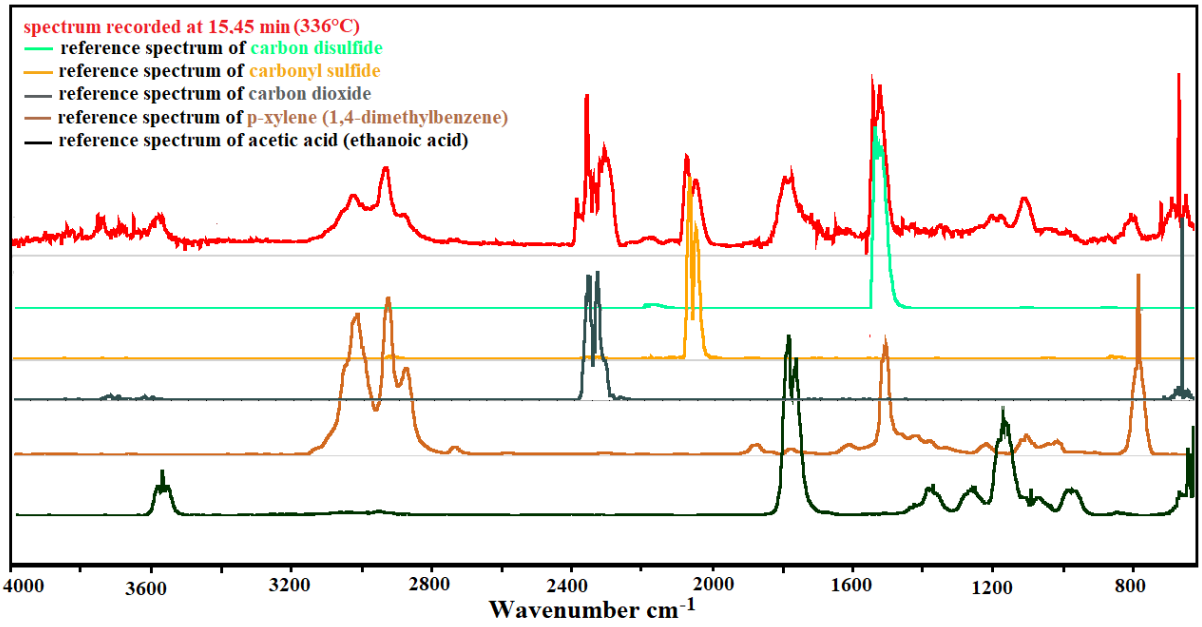

Figure S4. FTIR spectra of gaseous products obtained during the decomposition of **1P** recorded at 336 °C along with the reference spectra.

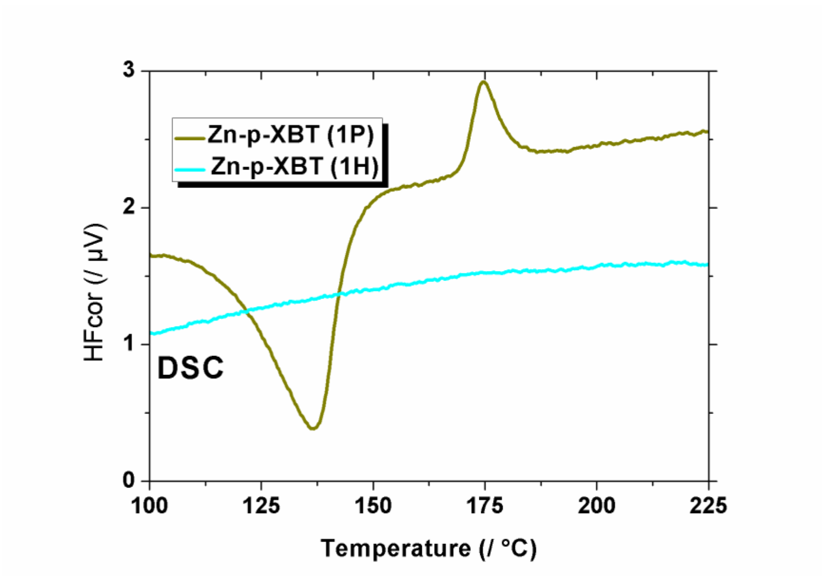

Figure S5. DSC plots of **1P** and **1H**.
